# Lessons learned: A neuroimaging research center's transition to open and reproducible science

**DOI:** 10.3389/fdata.2022.988084

**Published:** 2022-08-29

**Authors:** Keith A. Bush, Maegan L. Calvert, Clinton D. Kilts

**Affiliations:** Department of Psychiatry, Brain Imaging Research Center, University of Arkansas for Medical Sciences, Little Rock, AR, United States

**Keywords:** open science, reproducible neuroimaging, FAIR, preregistration, transition, neuroimaging

## Abstract

Human functional neuroimaging has evolved dramatically in recent years, driven by increased technical complexity and emerging evidence that functional neuroimaging findings are not generally reproducible. In response to these trends, neuroimaging scientists have developed principles, practices, and tools to both manage this complexity as well as to enhance the rigor and reproducibility of neuroimaging science. We group these best practices under four categories: experiment pre-registration, FAIR data principles, reproducible neuroimaging analyses, and open science. While there is growing recognition of the need to implement these best practices there exists little practical guidance of how to accomplish this goal. In this work, we describe lessons learned from efforts to adopt these best practices within the Brain Imaging Research Center at the University of Arkansas for Medical Sciences over 4 years (July 2018–May 2022). We provide a brief summary of the four categories of best practices. We then describe our center's scientific workflow (from hypothesis formulation to result reporting) and detail how each element of this workflow maps onto these four categories. We also provide specific examples of practices or tools that support this mapping process. Finally, we offer a roadmap for the stepwise adoption of these practices, providing recommendations of why and what to do as well as a summary of cost-benefit tradeoffs for each step of the transition.

## Introduction

The science of human functional neuroimaging has evolved dramatically in recent years, driven by a pair of emerging trends. First, neuroimaging science has grown ever more data intensive and computationally sophisticated. Indeed, with enhanced temporal and spatial resolution, newer generation multiband-accelerated 3-Tesla functional magnetic resonance imaging (fMRI) scanners acquire an order of magnitude more data per imaging session compared to earlier MRI systems (Demetriou et al., 2018). Advances in our understanding of myriad fMRI measurement confounds (Liu, 2017) necessitate the application of more complex preprocessing pipelines (Power et al., 2015), including multivariate modeling and machine learning (Mitchell et al., 2008; The Alzheimer's Disease Neuroimaging Initiative and Stern, 2010; Naselaris et al., 2011), as well as concurrent acquisition of additional biosignals (e.g., respiration) in order to identify the true neural processing representations underlying cognition (Jo et al., 2010). Second, emerging evidence indicates that functional neuroimaging findings are not reproducible. Complex pipelines infuse sufficient degrees-of-freedom into the practice of functional neuroimaging analysis that even highly experienced, critical practitioners routinely reach divergent conclusions – even for the same dataset and tested hypotheses (Botvinik-Nezer et al., 2020). Moreover, large-scale neuroimaging studies have revealed the true effect sizes for many common brain-behavior relationships, which are an order of magnitude smaller than previously believed (Poldrack et al., 2017; Marek et al., 2022), a finding with harrowing implications for recurrent error existing in the neuroimaging literature.

In response to a growing alarm within the neuroimaging community of the risks that these emerging trends pose to our ability to inform a mechanistic understanding of human behavior, neuroimaging scientists have developed many principles, practices, and tools to manage these risks. We group these best practices under four categories: experiment pre-registration, FAIR data principles, reproducible neuroimaging analyses, and open science. When implemented, these best practices enhance researcher confidence in their ability to control error – in both the research that they conduct as well as the scientific inferences they receive from the broader neuroimaging research community.

While there is growing recognition of the imperative of implementing these best practices there exits, in our opinion, a clear absence of how procedurally to accomplish this goal (Paret et al., 2022). This potential barrier to widespread adoption of open science practices is echoed for other fields (Kalandadze and Hart, 2022). In this work, we describe lessons learned from efforts to adopt these best practices within the Brain Imaging Research Center (BIRC) at the University of Arkansas for Medical Sciences over 4 years (July 2018–May 2022). For context as to the scale and complexity of these efforts, the BIRC is comprised of 5 full-time clinical and research faculty investigators as well as 6 full-time research and support staff. Over the course of this adoption process, the BIRC supported numerous National Institutes of Health, National Science Foundation, Department of Veterans Affairs, and Brain and Behavior Research Foundation funded neuroimaging projects. Moreover, the BIRC transitioned from a Philips 3T Achieva to a Siemens 3T MAGENTOM Prisma MRI scanning platform in the Fall of 2021.

We commence by providing brief summaries of the four categories of best practices. We then describe our center's scientific workflow (from hypothesis formulation to result reporting) and detail how each element of this workflow maps onto these four categories. We also provide specific examples of practices or tools that support this mapping process. Finally, we offer a roadmap for the stepwise adoption of these practices, providing recommendations, based on our personal experiences, of why and what to do as well as a summary of cost-benefit tradeoffs for each step of the transition. The goal of this work is to encourage and enable other human neuroimaging research groups to adopt similar strategies to better minimize replication failures and strengthen the inferences from this field of human neuroscience.

## Open and reproducible science solutions

### Preregistration

The practice of preregistration is the specification of a research hypothesis, and the methodology proposed to test this hypothesis, **prior** to gaining knowledge of the experimental outcome (Nosek et al., 2018). In practice preregistering a neuroimaging study entails (before the start of data-collection or before observing data in a secondary data analysis) compiling a detailed description of the experiment's hypotheses, data collection procedures, power analyses, data processing steps, and planned statistical modeling. This description then has two dissemination pathways. One path is to submit the description to an independent registry^1^ that publicly timestamps, indexes, and stores the information. Alternatively, the description may be submitted to an academic journal as a registered report (Nosek and Lakens, 2014; Chambers et al., 2015; Cockburn et al., 2020). Along this pathway, the description is formally peer-reviewed prior to the start of the research plan and, if accepted, has the advantage of being guaranteed publication once data collection and analysis are completed. Regardless of the dissemination pathway, preregistration seeks to explicitly separate confirmatory from exploratory analysis.

### FAIR data principles

The FAIR acronym^2^ stands for the data management principles of “findability,” “accessibility,” “interoperability,” and “reusability” (Wilkinson and Dumontier, 2016). Findable data are associated with globally unique and persistent identifiers, are richly described by metadata, and are registered in a searchable resource. Accessible data are accompanied by instructions for accessing the data and are stored in a protocol that is open and free to use by others. Interoperable data use a standardized knowledge representation which can be exchanged between applications and workflows for storage, processing, and analysis. Reusable data are described with accurate attributes and are released with clear usage licenses. FAIR principles seek to make data more widely actionable.

### Reproducible neuroimaging

Reproducible neuroimaging^3^ is a framework for annotating, versioning^4^, and analyzing neuroimaging data in order to promote the re-executability of published neuroimaging studies (Kennedy et al., 2019). This framework emphasizes the use of containerized image processing pipelines that enforce the reproducibility of multi-step transformations of neuroimaging data from a raw format (acquired directly by the MRI scanner) to a results representation (e.g., statistically significant regions of brain activation). Reproducible neuroimaging is built around the Brain Imaging Data Structure^5^ (BIDS) standard for formatting and storing neuroimaging data (Gorgolewski et al., 2016). The goal of this framework is to enhance the implementation of standards and approaches to best practice across diverse neuroimaging research groups.

### Open science

Open science is the practice of making research workflows, data, and results transparent and publicly accessible in order to improve scientific rigor, reproducibility, and replicability (Munafò et al., 2017). Open science promotes the use of open-source^6^, rather than proprietary software, the public repositioning of data preprocessing and analysis source code^7^, the public reposition of raw data^8^, and the publication of scientific findings as preprints^9^ as well as in open-access scientific journals that are freely accessible to the scientific and general public and which are dedicated to transparent and open reporting (Nosek et al., 2015). Open Science practice seeks to contribute to reducing the negative impact of intentional or unintentional questionable research practices on scientific integrity and reproducibility by promoting ready and widespread access to research methods, data, and outcomes (Yamada, 2018).

## A neuroimaging laboratory workflow

**Figure 1:**
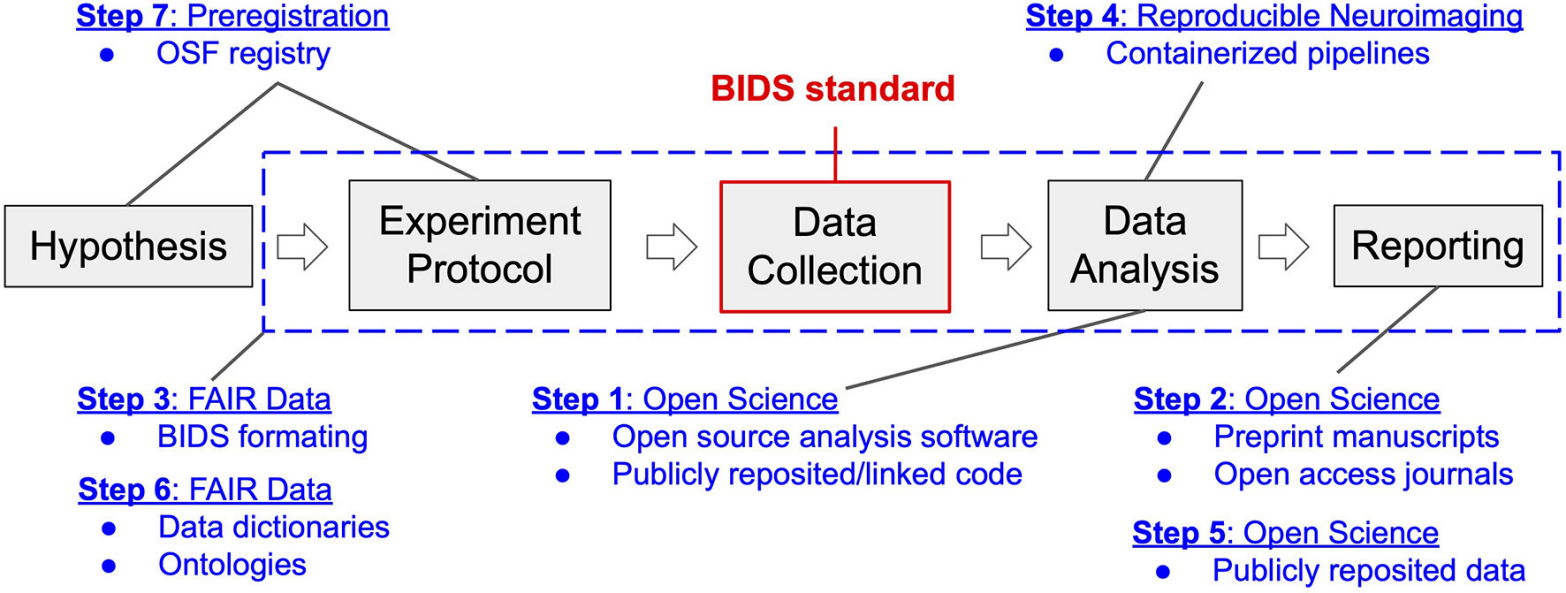
Neuroimaging workflow in the Brain Imaging Research Center in the context of transition to open and reproducible science best practices. Gray blocks represent the key steps in the workflow of a typical neuroimaging experiment, from inception to reporting. Blue text and dashed box indicate components of the four categories of open and reproducible best practices that map onto this workflow. Modules of transition (1–7) are reported according to the order (stepwise) of their implementation. The BIDS standard, highlighted in red, denotes the data structure around which the four categories of best practices revolve.

The research workflow utilized in our laboratory is akin to that deployed in the behavioral sciences, extended and modified to support our specific intent to identify the neural processing mechanisms of cognition and psychopathology (see Figure 1). We first formulate a hypothesis regarding a human brain-behavior relationship based on our theoretical understanding of the relevant extant literature. This hypothesis then informs the design of a neuroimaging experiment toward the goal of testing the falsifiable hypothesis. We define a neuroimaging experiment as a protocol (written procedure) for participant recruitment, screening, enrollment, and assessment, and that describes the methods of administration of common and/or novel behavioral tasks that purport to engage the neural processing network of interest. The experimental protocol also includes considerations specific to neuroimaging, such as MRI contraindications, as well as concurrent signal acquisitions that may be necessary to independently validate the veracity of the association of neural processing observations with the imposed processing demand or that are needed to isolate and remove imaging confounds and artifacts. Following institutional review board (IRB) approval of the protocol, we conduct imaging and non-imaging data collection and storage as per the protocol. Neuroimaging data analysis follows from stepwise best practice approaches to image processing and includes curation, minimal preprocessing (skull-stripping and spatial normalization of structural images as well as despiking, slice-time correction, motion correction, and transformation to the spatially normalized anatomic image for functional images). From these processed data we typically conduct whole-brain analyses to characterize between-group differences in neural activation responses to task demand (e.g., psychopathology vs. health control groups). Finally, we compile our findings into a manuscript and submit for peer-review. This workflow is denoted by the gray boxes and arrows depicted in Figure 1.

Prior to July 2018, our center's workflow had not incorporated best practices for open and reproducible science. Our data was stored in non-standard formats (different even between principal investigators within the same center). Our experimental paradigms and processing pipelines were written in closed-source programming languages (Presentation and Matlab), which were maintained locally without comprehensive version control that could not be reviewed or linked to manuscripts. Also, our center had never released a manuscript preprint and had only occasionally published in open-access journals. This then-common approach represented the starting point for the planned transition.

## The transition plan

In July of 2017, compelled by concerns surrounding the neuroimaging research reproducibility crisis inspired by Eklund et al. (2016) as well as growing concerns that our failure to be open and transparent in our work would lead to costly errors or replication failures in the future, our imaging center committed to transitioning to the use of best practices in open and reproducible science. We committed to a lengthy period of literature review through which we identified the four categories of best practices, described above. We also identified critical tools and resources that we would need in order to achieve best practice outcomes. Across the four categories, we identified seven modules of work that would be necessary to achieve a transition to best practices. We qualitatively rank-ordered these modules according to the expected implementation difficulty given the skillsets and resources available within our imaging center as well as the expected benefits to the center once implemented. We then laid out a stepwise, one step per module, plan for the center's transition to best practices, depicted in Figure 1, prioritizing those modules with the highest perceived benefits to costs ratio. We enumerate this plan below and provide details of the reasoning behind the proposed sequencing of the steps as well as our appraisal of accrued costs and benefits.

### Open science: Transition to open-source analysis code and public code reposition (step 1)

Prior to July 2018, Matlab (a proprietary language associated with annual licensing costs) was the primary programming language used within our center. Our center also had no standardized software maintenance and version control practices. Our first task was to integrate our legacy proprietary code base with git, a free and open source version control system for tracking software changes and updates (Chacon and Straub, 2022). Further, we started publicly repositing much of our legacy source code on Github (e.g., see https://github.com/kabush/CTER) and commenced linking relevant source code within manuscripts submitted for peer-review. Our second task was to re-implement our analysis pipelines in an open-source language, Python. Due to in-house computer science expertise, this module of work had relatively low implementation costs for our center. However, the costs of executing this transition step would vary by laboratory depending on one's access to computer science or software engineering expertise. For newly forming neuroimaging labs, we recommend *a priori* adoption of open-source languages, such as Python and R, which have large and growing neuroimaging processing and analysis software ecosystems (Brett et al., 2009; Muschelli et al., 2019). While implementation costs will vary, the benefits of implementing this transition step were perceived to be high. Versioning and reposition tools such as git and Github facilitate collaboration among teams of software developers. They also promote the sharing of source code externally (e.g., within manuscripts or amongst peers). Specific to the academic setting, code repositories assist in the transfer of software projects between training personnel, particularly when trainee turnover is high (~4 years for doctoral students) relative to the lifespan of the project, thus minimizing the cost of lost intellectual capital. Public sharing of code also facilitates the detection of software or processing errors and thereby facilitates greater rigor and reproducibility of scientific analyses (Ince et al., 2012) and promotes trust in research outcomes (Stodden, 2011).

### Open science: Transition to preprinting manuscripts prior to peer-review (step 2)

The easiest open and reproducible science practice to implement is the publishing of preprints of scientific manuscripts prior to peer-review. Many preprint servers exist on which to disseminate one's work (e.g., arXiv, bioRxiv, psyRxiv). The direct costs of preprinting are minimal and reflect the effort of uploading the manuscript to the preprint server and a short time delay (typically several days) required for moderators to review the manuscript prior to public reposition (and assignment of a digital object identifier, DOI). The indirect cost of preprinting stems from the potential risk of publicly exposing a flaw in one's work, rather than the flaw being detected and fixed privately within the peer-review process (Kaiser, 2017). The benefits of preprinting arise from the ability to rapidly share one's work with the public and receive feedback while maintaining ownership and attribution of the ideas and results contained within the manuscript (Sarabipour et al., 2019). An additional indirect benefit of preprinting is the potential detection of flaws prior to formal peer-review as was demonstrated by a highly publicized exchange of preprinted manuscripts analyzing task data within a large, publicly-available neuroimaging dataset (Bissett et al., 2020, 2021; Garavan et al., 2020). Finally, many science publishers now allow manuscript submission to their peer-review process directly from preprint servers, significantly reducing the time and effort of submission^10^ With a favorable benefit-cost ratio, our center has transitioned to preprinting all manuscripts prior to peer-review.

### FAIR data: Format in-house data to the BIDS standard (step 3)

The single most important module that we implemented throughout the transition process was formatting of our center's raw data in BIDS (Gorgolewski et al., 2016). BIDS is a new standard (circa 2016) for organizing neuroimaging, psychophysiological, behavioral, and demographic data to enable ease-of-sharing and software interoperability across datasets and imaging technologies. The person-hour cost of constructing our formatting infrastructure was formidable. Learning the BIDS specification, implementing software pipelines to map the data (e.g., see https://github.com/kabush/CTER2bids), and validating that the resultant mappings met the BIDS standard consumed many months of effort across multiple imaging center team members. Indeed, despite in-house computer science expertise, this module of work was the single most costly step in the transition process. The benefits of mapping our data to BIDS, however, far exceed the costs. First, our center now has access to BIDS-Apps (Gorgolewski et al., 2017), which are containerized data processing and analysis tools built to operate seamlessly on validated BIDS datasets (see Steps 4 and 5). Moreover, custom analysis code can now be written in-house to operate on the BIDS standard, which dramatically reduces the number of variables, e.g., naming, structuring, and formatting, that previously pertained to adapting in-house code to projects across PIs within our imaging center.

### Reproducible neuroimaging: Transition to containerized preprocessing pipelines (step 4)

Immediately following our transition to BIDS, we replaced our in-house minimal image preprocessing pipeline with fMRIPrep (Esteban et al., 2019), a well-validated, widely used, and robust pipeline for functional neuroimaging data built upon the BIDS-Apps framework. We also implemented in-house data quality assurance processes based upon MRIQC (Esteban et al., 2017), a BIDS-App tool for deriving quantitative measures of image quality to support automated, data-driven inclusion/exclusion decisions for statistical analysis. The implementation cost of this step was low, requiring the installation of Singularity^11^, an engine for executing containerized software, on our high-performance computer system (a 96 core Dell PowerEdge R630 server running the CentOS operating system) as well as the programming of simple interfaces to execute these tools on our data (e.g., see https://github.com/kabush/birc-preproc). The benefit of implementing this step is that our minimal image preprocessing pipeline is now both well-validated and reproducible, and thus of high value to controlling error and the ultimate goal of developing transparent, reproducible workflows.

### Open science: Public reposited data (step 5)

An additional benefit of mapping our datasets to BIDS is the ease with which we can now reposit and publicly share our neuroimaging data on-line *via* OpenNeuro (Markiewicz et al., 2021). All data uploaded to OpenNeuro must pass BIDS validation and, once validated, receives a digital object identifier (DOI). Thus, OpenNeuro reposited data comports with the four FAIR principles of findability, accessibility, interoperability, and reusability. Assuming that appropriate legal and ethical authorizations have been obtained, the effort cost of publicly repositing de-identified data (given that it is already formatted to the BIDS standard) on OpenNeuro is negligible. The benefits of repositing data on OpenNeuro are twofold. First, repositing data publicly allows the dataset to be linked to the data availability statements of manuscripts when submitting to peer review, thereby giving reviewers direct access to the raw materials that support the scientific inferences. Second, permanently repositing BIDS formatted data yields an immutable record of the institutional knowledge that went into acquiring the dataset. This maintains the dataset's integrity over time even as the staff and trainees that built them transition out of the center.

### FAIR data: Establish data dictionaries (step 6)

The BIDS standard establishes the structure of neuroimaging data but not its content (Kennedy et al., 2019). However, the FAIR data principle of interoperability aims to promote semantically rich datasets in which the meaning of each reported data element is defined (Wilkinson and Dumontier, 2016). This semantic annotation is achieved by linking each data element to a unique identifier from a publicly available, consistent, and controlled vocabulary (i.e., ontology). Ontologically-augmented BIDS datasets have been termed ReproBIDS (Kennedy et al., 2019). Due to restrictions posed by the COVID-19 pandemic and the replacement of our core 3T MRI system, our ability to enroll, assess, and scan subjects was limited. We used this time to construct a center-wide data dictionary which assigns both a publicly accessible ontology and unique ontological identification number to the subscale-level for all participant assessment instruments as well as neuroimaging modalities, psychophysiological modalities, and behavioral tasks for all current and planned studies within our center. The person-hour cost of this data dictionary construction was substantial and the short-term benefits have been limited to an important understanding of the quality of data collected in-house. However, the long-term benefits of ReproBIDS, for example through the use of machine learning to conduct automated *post-ho*c mining of neuroimaging datasets (Wilkinson and Dumontier, 2016), are anticipated to be large.

### Preregistration: Open science framework registry (step 7)

In the final transition step, our center is migrating to preregistration of upcoming research studies using, where appropriate, either the Open Science Framework registry or clinicaltrials.gov. We also now encourage Center investigators to budget – in all upcoming research proposals – initial effort related to formally preregistering the study's aims. At present we have multiple study preregistrations in development and review. As preregistration entails assembling a document (study abstract and methods) of similar depth and detail as would be necessary to submit a manuscript for consideration of publication as a journal article, preregistration can be thought of as front-loading the effort of writing a journal manuscript without the results and conclusion. The Open Science Framework registry provides a template^12^ to guide neuroimaging scientists through the technical details of best practices reporting of both fMRI and psychometric data collection. Therefore, the absolute effort cost of preregistration is low, but the distribution of cost is disproportionately front-loaded to the study's inception. The potential benefits of preregistration, however, are manifold (Nosek et al., 2019). By pre-specifying all planned aspects of a study, preregistration clarifies planned versus unplanned data analyses which mitigates questionable research practices (Yamada, 2018) such as “hypothesizing after results are known” (Kerr, 1998) (aka HARKing) and p-hacking (Simmons et al., 2011) that promote publication bias and increase the prevalence of false-positive findings in the literature. Preregistration also incentivizes the publication of null findings (Allen and Mehler, 2019), which improves our long-term understanding of true effect sizes derived *via* meta-analyses (Ioannidis, 2005).

## Summarizing the costs and benefits of transition

Perhaps the greatest barrier to making the transition to open and reproducible science is the uncertainty surrounding the costs and benefits of the process. To assist others in planning their own transition we have summarized (see Table 1) the cost-benefit tradeoff for each step of the road-map described in Section “ The Transition Plan”. For simplicity and clarity, we have reduced the costs and benefits to a 3-point Likert scale of {Low, Medium, High} that approximates the authors' post-hoc estimate of the time and effort that the imaging center invested (or will invest) in making the transition (Cost) as well as the amount of time saved, or is anticipated to be saved (Benefit) in combination with end-goal benefits such as increased perceived trust in the rigor and reproducibility of the science (both internally and externally) as well as expedited dissemination of findings. This cost-benefit summary has value to neuroimaging scientists who want to weigh a point of entry into the transition process or wish to migrate to adopting those open science principles of the total that best suit their research focus.

**Table 1:** Summary of open and reproducible neuroimaging transition costs-benefits analysis.

	**Category**	**Description**	**Cost**	**Benefit**
Step 1	Open science	Open-source analysis software and public repositing of analysis source code	Low	High
Step 2	Open science	Preprint manuscripts	Low	Medium
Step 3	FAIR data	Map data to the BIDS standard	High	High
Step 4	Reproducible neuroimaging	Containerized preprocessing pipeline	Low*	High
Step 5	Open science	Public data reposition	Low*	Medium
Step 6	FAIR data	Data dictionaries	High	Low**
Step 7	Preregistration	Open science framework registry	Medium***	Medium****

* Assumes BIDS mapping was previously completed (see Step 3).

** Based on estimated short-term benefits. Long-term benefits may be much larger than those observed.

*** Cost is temporally shifted from post-hoc to a priori effort.

**** In progress. Only anticipated benefits listed.

## Final thoughts on the transition

The Brain Imaging Research Center at the University of Arkansas for Medical Sciences has expended significant effort over the past 4 years incorporating best open and reproducible practices into our scientific workflow. What began as a process to eliminate scientific risk – the risk of serious scientific errors, false-positive claims, replication failures, or losses of critical institutional knowledge – evolved into a pursuit of seamless and efficient scientific inquiry in which each step of the scientific process, from project inception to results reporting, is thoughtfully, rigorously, and transparently documented and shared. This is, in our opinion, both the scientific ideal and mandate.

However, we acknowledge that migrating a neuroimaging laboratory to best practices in open and reproducible science is a non-trivial undertaking associated with substantial initial costs of effort to be borne by individuals already engaged in challenging, time-consuming work. During transition, the lab's record of research productivity will suffer. Projects will need to be implemented in both the legacy and upgraded workflows so that scientific products, the lifeblood of the lab, continue to be generated on a regular basis. Each member of the lab will likely need to invest in learning additional programming, version control, and data management skills as well as acclimate to working with unfamiliar naming conventions and directory structures mandated by the BIDS standard. The entire process may be frustrating and intimidating. We also acknowledge that while many argue for the benefits of open science practices to investigators and science itself (Frankenhuis and Nettle, 2018), others argue that derived benefits are specious and even sinister (Mirowski, 2018) and that some open science practices may create a paradoxical outcome of public distrust and misinformation (Besançon et al., 2021).

The purpose of this work is to provide a brief experience-based depiction of the terrain that a lab will cover in their own migration to this responsible commitment to open and reproducible neuroimaging science. We have condensed a large and rapidly growing literature down to four categories of tools and practices that comprise the open and reproducible science ecosystem. For new investigators starting their own labs, we strongly encourage incorporating these practices into the lab's workflow from Day 1. For established labs, such as ours, we provide a 7-step model of transition that prioritizes practices with the lowest costs and highest benefits. Of the initial 5 recommended steps, only the transition to BIDS format imposes a high cost, but it also bears the greatest number of benefits and thus, we argue, is the single most important step a lab should undertake. We hope that these lessons learned will inform your own decision as to when and where, not if, you will start the transition.

## Data availability statement

The original contributions presented in the study are included in the article/supplementary material, further inquiries can be directed to the corresponding author.

## Author contributions

Conception, manuscript preparation, and revisions: KB, MC, and CK. All authors contributed to the article and approved the submitted version.

## Conflict of interest

The authors declare that the research was conducted in the absence of any commercial or financial relationships that could be construed as a potential conflict of interest.

## Publisher's note

All claims expressed in this article are solely those of the authors and do not necessarily represent those of their affiliated organizations, or those of the publisher, the editors and the reviewers. Any product that may be evaluated in this article, or claim that may be made by its manufacturer, is not guaranteed or endorsed by the publisher.

## Footnotes

^1^ https://www.cos.io/initiatives/prereg

^2^ https://www.go-fair.org/

^3^ https://www.repronim.org/

^4^ https://www.datalad.org/

^5^ https://bids.neuroimaging.io/

^6^https://cran.r-project.org and https://python.org

^7^ https://github.com

^8^https://osf.io/ and https://openneuro.org/

^9^ https://www.biorxiv.org/

^10^ https://www.biorxiv.org/submit-a-manuscript

^11^ https://sylabs.io/guides/3.5/user-guide/

^12^ https://osf.io/6juft/
